# The Influence of Exercise on Oxidative Stress after Spinal Cord Injury: A Narrative Review

**DOI:** 10.3390/antiox12071401

**Published:** 2023-07-08

**Authors:** Grazia Maugeri, Alessandra Amato, Martina Sortino, Velia D′Agata, Giuseppe Musumeci

**Affiliations:** 1 Section of Anatomy, Histology and Movement Sciences, Department of Biomedical and Biotechnological Sciences, University of Catania, 95123 Catania, Italy; graziamaugeri@unict.it (G.M.); alessandra.amato@unict.it (A.A.); martinasortino97@gmail.com (M.S.); vdagata@unict.it (V.D.); 2 Research Center on Motor Activities (CRAM), University of Catania, 95123 Catania, Italy

**Keywords:** spinal cord injury, exercise, reactive oxygen species

## Abstract

Spinal cord injury (SCI) is an irreversible disease resulting in partial or total loss of sensory and motor function. The pathophysiology of SCI is characterized by an initial primary injury phase followed by a secondary phase in which reactive oxygen species (ROSs) and associated oxidative stress play hallmark roles. Physical exercise is an indispensable means of promoting psychophysical well-being and improving quality of life. It positively influences the neuromuscular, cardiovascular, respiratory, and immune systems. Moreover, exercise may provide a mechanism to regulate the variation and equilibrium between pro-oxidants and antioxidants. After a brief overview of spinal cord anatomy and the different types of spinal cord injury, the purpose of this review is to investigate the evidence regarding the effect of exercise on oxidative stress among individuals with SCI.

## 1. Introduction

Spinal cord injury (SCI) is a potentially devastating condition for patients that can be caused by non-traumatic or traumatic events [1]. Patients with SCI may sustain multiple sensorimotor deficits. These can include full or partial paralysis of muscles below the lesion; muscle spasms; spasticity; neuropathic pain; and bowel, bladder, and sexual dysfunctions. Deficits in neurological function have significant impacts on the metabolism and can lead to subsequent metabolic-related disease risk (e.g., type 2 diabetes and cardiovascular disease) in SCI patients. Subjects with high-level injuries have manifested reduced glucose tolerance, greater insulin resistance, impairment of lipid profiles, decreased bone density and muscle mass, and thermoregulatory alteration leading to periods of hypothermia [2].

The worldwide incidence of traumatic SCI is ~26.5 cases per 1,000,000 inhabitants, and most cases are males (68.3%) [3]. SCI can be divided into primary and secondary phases [4]. The latter is characterized by multifaceted pathological events that may last for months and years [5,6] It is well-known that oxidative stress contributes to a harsh post-injury microenvironment, causing further cell death by necrosis and apoptosis. Therefore, counteracting reactive oxygen species (ROS) generation and oxidative stress are essential strategies for SCI treatment. Physical exercise is an indispensable element in ensuring a correct lifestyle, and an inverse dose–response link exists between volume of physical activity and all-cause mortality [7,8]. In accord, regular exercise promotes psychophysical well-being, preventing and managing disorders of the osteomuscular, cardiovascular, endocrine, and immune systems and the onset of potential cancer [9,10,11]. In the nervous system, physical exercise induces neurogenesis and brain plasticity, enhancing cognitive and motor functions. It promotes axonal growth, induces phenotypic changes in peripheral structures, and positively affects the levels of neurotrophins such as brain-derived neurotrophic factor (BDNF) [12,13]. Furthermore, exercise has been demonstrated to improve insulin resistance, adipose fuel metabolism, inflammation, and epigenetic factors by preventing and mitigating the impacts of secondary metabolic diseases related to SCI [14].

According to the 2020 World Health Organization (WHO) guidelines, adults should perform 150–300 min of weekly moderate aerobic exercise or 75–150 min of vigorous-intensity physical activity per week [15]. On the other hand, physical exercise at higher intensities is associated with ROS overproduction and/or alteration in the antioxidant defense systems, leading to oxidative stress [16]. Considering the harmful role of oxidative stress in SCI, our objective is to review the influence of exercise on it after SCI. Thus, this review aims to provide an overview of spinal cord anatomy and the types of SCI and discuss studies present in the literature regarding the effects of exercise on the oxidative/antioxidative ratios of SCI patients.

## 2. Overview of Spinal Cord Anatomy

**Figure 1:**
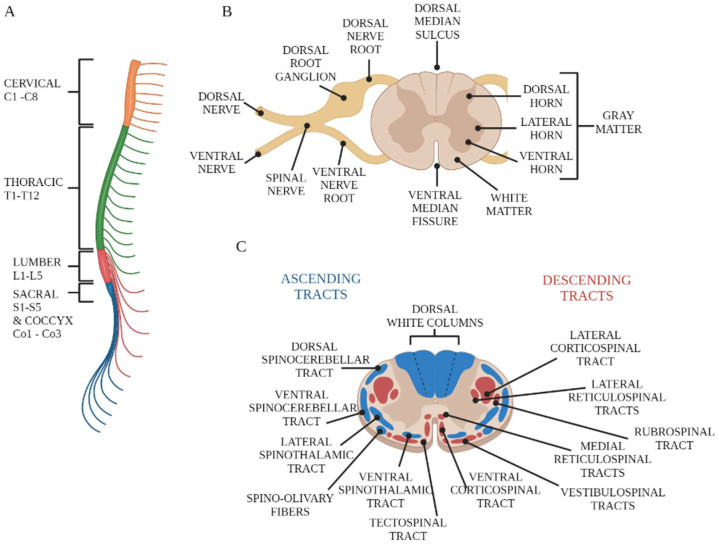
Spinal cord anatomy. (**A**) Neuromeres of spinal cord. (**B**) Organization of spinal cord into gray matter, containing neuronal cell bodies, and white matter, (**C**) containing myelinated axons organized in ascending or descending tracts (created with BioRender.com, https://app.biorender.com, accessed on 26 June 2023).

The central nervous system (CNS) consists of two parts: the brain and the spinal cord. The spinal cord, following the brainstem, represents the most caudal portion of the central nervous system. The spinal cord is a long, cylindrical extension contained and protected within the vertebral canal. It is one-quarter the length of the individual’s height, averaging 42.3 cm in males and 38.9 cm in females [17]. The spinal cord has two characteristic enlargements corresponding to the emergence of the nerve roots for the respective brachial and lumbosacral plexuses, which innervate the muscles of the upper and lower limbs, respectively. Inferiorly, the spinal cord gradually decreases in size and terminates at the body of the second lumbar vertebra in the medullary cone, which in turn extends into a connective filament, attached dorsally to the first coccygeal vertebra, called the filum terminale. The pearly white surface of the spinal cord shows a deep longitudinal fissure in the anterior midline; this is the anterior median fissure. In contrast, the posterior surface shows a shallow groove in the midline; this is the posterior median fissure. In addition, shallow depressions known as anterolateral and posterolateral grooves are present on both sides. The ventral (motor) roots of the spinal cord emerge from the anterolateral groove, while the dorsal (sensory) roots of the spinal cord penetrate the posterolateral groove. The spinal cord can be divided into a series of transverse segments (or neuromeres) that are grouped into regions. The name of each region corresponds to that of the portion of the spinal column from which the corresponding spinal nerves emerge. Thus, it is possible to identify the cervical region (C1–C8), the thoracic region (T1–T12), the lumbar region (L1–L5), the sacral region (S1–S5), and the coccygeal region (Co1–Co3) (Figure 1A). The first spinal nerve exits between the foramen magnum and the first cervical vertebra; all other spinal nerves exit progressively from the successive intervertebral spaces. The transverse section of the spinal cord is formed by a gray substance internally and a white substance arranged in the periphery [18] (Figure 1B). The gray substance is shaped like an H or a butterfly and is crossed by the central canal. This canal, lined with ependymal cells, contains a small amount of cerebrospinal fluid and is in continuity cranially with the fourth ventricle, while caudally, it exceeds the *filum terminale* by a few millimeters, terminating in a blind end. The gray substance contains the cell bodies of the spinal cord neurons, glial cells, and vessels. Its H shape derives from the presence of two lateral masses (horns) arranged in a “C” shape, with the concavity facing sideways and joined by a transverse section (gray commissure) crossed by the central canal. The dorsal horns receive sensitive information that enters the spinal cord through the dorsal roots of the spinal nerves. The ventral horns contain the pyrenophores of the motor neurons that send axons through the ventral roots of the spinal nerves to terminate on striated muscles. The lateral horns, located only in the neuromeres from C8 to L2, contain visceral preganglionic motor neurons that project to the sympathetic ganglia. White matter is the collection of myelinated and unmyelinated nerve fibers that run through the spinal cord. The amount of white matter will decrease in a craniocaudal direction, contrary to the gray matter, whose occupied space will increase. The white matter of the spinal cord comprises funicular fibers with a longitudinal course; they are organized into dorsal (or posterior), lateral, and ventral (or anterior) columns (Figure 1C). Each column is dedicated to the transport of specific information. In particular, the dorsal columns transport ascending sensory information from somatic mechanoreceptors. The lateral columns comprise axons that travel from the cerebral cortex to contact spinal motor neurons. The ventral (i.e., ventrolateral and anterolateral) columns carry both ascending pain and temperature information and descending motor information [19].

## 3. Spinal Cord Injuries

**Figure 2:**
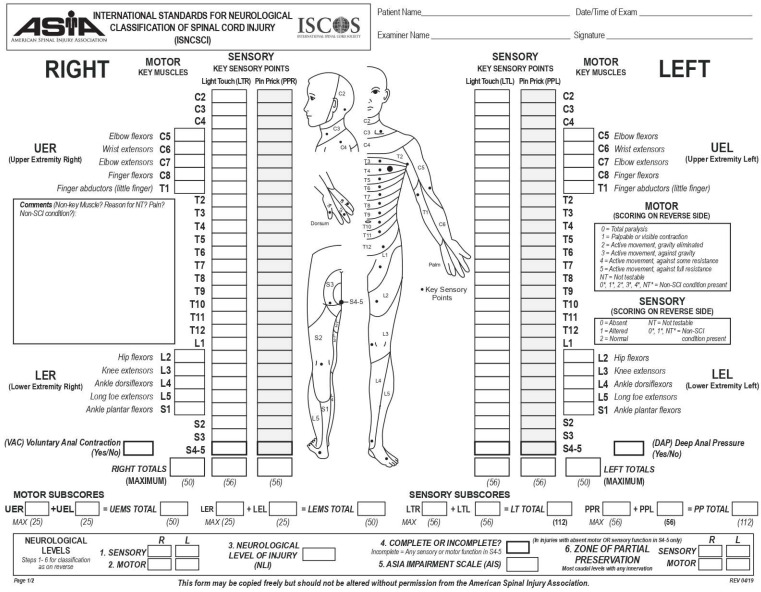
ISNCSCI Worksheet. https://asia-spinalinjury.org/international-standards-neurological-classification-sci-isncsci-worksheet/ (accessed on 1 June 2023).

A dying lioness, depicted on a stone panel from the Assyrian Palace of Ashurbanipal and dating from around 650 BC, is the earliest known graphic example of SCI. The panel shows a lioness trying to crawl with her lower extremities paralyzed due to arrows running through her back. SCI is a devastating neurological condition that can significantly reduce quality of life and has a tremendous socioeconomic impact on affected individuals and the health care system. The term SCI is used to refer to damage to the tight bundle of cells and nerves through which the spinal cord (1) receives signals from the body and sends them to the brain and (2) receives motor commands from the brain and sends them to the rest of the body [20]. This damage is characterized by several temporary or permanent symptoms such as paralysis, paresthesia, spasticity, pain, weakness, changes in sexual function, and loss of bladder and bowel control [21]. The life expectancy of SCI patients highly depends on the gravity and localization of the lesion, which can be incomplete or complete, leading to the loss of sensory and/or motor function below the level of the injury [22]. The severity of SCI is classified into different grades (Table 1) based on the International Standards for Neurological Classification of Spinal Cord Injury (ISNCSCI) (Figure 2) developed by the American Spinal Injury Association (ASIA). A complete SCI is characterized by the loss of all motor and sensory functions, including sacral roots, distal to the site of injury (Grade A). Injuries classified as B through E on the AIS are incomplete and have some degree of retained motor or sensory function below the site of injury. Patients affected by Grade E injuries show normal motor and sensory functions but still may have irregular reflexes or other neurological phenomena [22].

SCI can be due to a non-traumatic or traumatic event. In the former case, damage to the spinal cord occurs due to a degenerative disease, infection, or tumor. Conversely, traumatic damage is more common and occurs when a traumatic impact fractures or dislocates the vertebrae [23,24,25]. The pathophysiology of SCI comprises an initial primary injury followed by a secondary phase of injury in which oxidative stress plays a key role. Primary injury will result immediately at the time of the initial impact. The mechanical insult on the spinal cord will harm resident tissue, and the bony or disk fragments will provoke damage or transection of the spinal cord and the surrounding structures. This phase is characterized by local hemorrhaging within the spinal cord tissue and by edema. In fact, following the traumatic injury, blood–spinal cord barrier permeability is increased, causing the entry of osmotically active substances into the injury site [22]. The bleeding that characterizes the early time of SCI is later interrupted, causing hypoxia and ischemia, which concur with the initiation of secondary-phase SCI [26,27]. The secondary injury is a multicascade of pathomechanisms occurring in the hours, days, and weeks following the primary injury and not only involves the site of the initial primary injury but also spreads to adjacent tissue. These events include alterations in electrolytes; production of ROSs; apoptosis and necrosis; increases in inflammatory factors such as tumor necrosis factor (TNF)-α, cytokines (IL-1α and IL-1β), and transforming growth factor (TGF)-α; the release of nitric oxide and glutamate; glial scar formation (gliosis) of astrocytes; significant increases in the frequency of chemokines; human growth-regulated oncogene/keratinocyte chemoattractant (GRO/KC); and macrophage inflammatory protein-1(MIP-1α) [28,29,30].

## 4. Oxidative Stress in SCI

The severity of SCI is strictly linked to the balance between the production of ROSs and the ability of the antioxidant system to detoxify these reactive chemical species [31,32]. ROSs are unstable molecules, containing oxygen, that easily react with other molecules in a cell. These comprise oxygen-free radicals (e.g., superoxide, hydroxyl radicals, lipid radicals) and non-radicals (e.g., hydrogen peroxide, lipid peroxide, peroxynitrite) (Table 2). ROSs are controlled by antioxidant mechanisms that include enzymatic and non-enzymatic molecules. Low-molecular-weight non-enzymatic antioxidant compounds include glutathione, vitamins C and E, β-carotene, and uric acid. Conversely, antioxidant enzymes include superoxide dismutase (SOD), catalase, glutathione reductase, and glutathione peroxidase [27].

Under physiological conditions, mitochondria represent the major source of cellular ROSs, since they use ~90% of the oxygen employed by cells during oxidative phosphorylation. However, SCI is characterized by mitochondrial dysfunctions that furtherly increase ROS generation [33]. ROSs are also generated by microglia and leukocytes that, after injury, show a significant increase in oxygen consumption and synthesize ROSs primarily with the enzyme systems of nicotinamide adenine dinucleotide phosphate (NADPH) with myeloperoxidases, cyclooxygenase, and xanthine oxidase [34]. Under normal conditions, ROSs regulate several biochemical processes, such as cell differentiation [35], neurogenesis [36], antioxidant gene expression [37], and the immune system [38]. In contrast, following SCI, increased ROS formation can lead to damage to lipids, proteins, nucleic acids, cells, and tissues, inducing necrosis and apoptosis [39,40]. Spinal cord tissue is particularly sensitive to oxidative damage [27]. In early SCI studies conducted in rodent models, ROS overexpression was observed from 1 to 24 h after injury [41,42,43,44,45,46]. In a contusion model of SCI in rats, a significant expression of oxidative stress markers was found from 3 h post-injury. Moreover, that expression was rapidly expanded within gray and white matter at up to 72 h post-injury [47]. Other studies performed in SCI rat models demonstrated the presence of high levels of ROSs at up to at least 10 days post-injury [48,49,50]. High levels of oxidative stress-related proteins, such as glutathione S-transferase Yb-3 and apolipoprotein A-I precursor peroxiredoxin-6, were detected at 24 h following acute spinal cord contusion in rats [51]. Another study undertaken in a rat model of moderately severe contusion SCI showed that peroxynitrite rapidly accumulated at early time points and an important increase was sustained up to 1 week after injury [52]. High levels of ROSs were also detected in blood samples from patients with cervical SCI for up to 7 days [53]. However, SCI also results in long-lasting oxidative stress. In fact, in the blood of subjects with SCI, high levels of oxidative stress biomarkers have significantly increased in the first year after an acute SCI. On the other hand, one month after the injury, levels of antioxidants, such as total and oxidized glutathione, different carotenoids, and α-tocopherol, were reduced by 19% to 71% as compared to control subjects [54]. This result is in line with previous evidence showing that SCI causes a significant decrease in the antioxidant content of the spinal cord compared to significant increases in oxidative stress markers [46,55,56].

## 5. The Influence of Exercise on Oxidative Stress in Individuals with SCI

SCI is highly complex from its pathophysiology to its management, and to date, there is no resolving clinical therapy. A robust portion of the literature has demonstrated that exercise has several beneficial effects after SCI.

Exercise strengthens paralyzed muscles and promotes the recovery of motor functions [57,58]. In an intact spinal cord, exercise dynamically modulates adult neurogenesis mediated by the ACh and GABA neurotransmitters [59]. In SCI rodent models, endurance exercise has promoted axonal regeneration via hormonal mechanisms, DNA methylation, and BDNF expression [60,61,62]. Moreover, exercise has increased myelination and restoration of serotonergic fiber innervation to the lumbar spinal cord, promoting the survival of grafted neural stem cells via IGF-1 [63]. It has also improved neuroplasticity and restored motor and sensory functions in SCI patients, also affecting the secondary consequences of SCI, such as chronic inflammation and cardiometabolic syndrome [64,65,66,67].

In able-bodied individuals, physical exercise leads to an immediate increase in oxidative stress levels, followed by high antioxidant enzyme activity [68,69,70]. Similarly, in SCI subjects, exercise induces the increase in oxidative stress as the first response, but generally, this increment is balanced by a progressive activation of antioxidative activities such as the production of SOD and GPx glutathione peroxidase (GPx) [70] or a reduction in oxidized lipid levels. In this way, exercise trains the ability to rebalance the antioxidative to oxidative activity ratio. Indeed, it has been demonstrated that ROS production during exercise, through a feedback mechanism, activates the process of antioxidant enzyme production through cell-signaling processes [71]. Moreover, people who train have higher glutathione concentrations at rest and lower resting concentrations of glutathione disulfide and malondialdehyde (markers of oxidative stress) [72,73,74]. In contrast, a sedentary lifestyle, without the intake of adequate nutrients in quantity and quality, will result in increases in circulating glucose and fatty acids that will induce, through the mitochondrial electron transport chain, excess production of ROSs [75]. Thus, there is already evidence that, in non-pathologic subjects, fitness level, often assessed as the maximum rate of oxygen employed during exercise, strongly influences antioxidant capacity. SCI subjects generally have lower maximum oxygen consumption (VO2 max) than normal subjects, as it turns out that less muscle mass is activated during exercise. In addition, the atrophy characteristic of SCI leads to progressive loss of lean mass, which also has intrinsic alterations, such as reduced mitochondrial oxidative capacity [76]. This condition, certainly depending on the type and level of injury, predisposes this population to chronic increases in oxidative stress [77]. In addition, subjects with SCI and sedentary lifestyles seem to have higher levels of lipid peroxides both at rest and in response to a single exercise test. From this evidence follows the hypothesis that exercise may influence the management of oxidative stress even in SCI subjects. However, to date, the studies in the literature that have examined the effects of training protocols on oxidative stress in humans with SCI are few and heterogeneous in their results, as several open questions exist. First of all, there is one regarding the differential effects of different types, durations, and intensities of exercise: for example, between aerobic, anaerobic, and combined aerobic–anaerobic training. Another open question is that of different metabolic reactions to chronic or acute exercise. When aerobic training is repeated over time and becomes “chronic”, it will result in reduced LDL oxidation, but a single exercise to exhaustion (acute) is associated with a progressive increase in LDL. Thus, regular training could give benefits in managing the oxidative/antioxidative ratio, but intense and prolonged exercise inappropriate to a subject’s characteristics and fitness level could result in excess ROS production in various tissues, increasing oxidative stress [78,79]. For these and other reasons described above, we wanted to discuss in this section the current studies present in the literature (Table 3) that have investigated the effects of any exercise protocol (chronic or acute) by evaluating oxidant and antioxidant markers in subjects with SCI to understand what the clear results are and, above all, what the questions not yet investigated regarding the regulation effects of the oxidative stress of physical exercise in subjects with SCI are.

Van Duijnhoven et al. [80] and Goldhardt et al. [81] (Table 3) have evaluated the chronical and acute effects, respectively, of associating functional electrical stimulation (FES), with aerobic training protocols, with the oxidative stress of subjects with SCI. This research hypothesis is based on the fact that it has already been demonstrated that muscle activation by FES in this population immediately increases oxygen consumption [82]. Van Duijnhoven et al. hypothesized that a single exercise with FES would immediately alter oxidative stress but improve the antioxidative capacity when the exercise became chronic. Therefore, for 8 weeks, they trained subjects with chronic SCI by applying gradual electrical stimulation during leg cycle ergometer exercise with a constant rpm frequency (Table 1 reports the training parameters) but did not find a difference in the concentrations of oxidative and antioxidative markers between before and after those 8 weeks. Therefore, the intensity of this training method appears to be ineffective in increasing the antioxidant capacities of subjects with SCI, but subjects should be able to tolerate it without an increase in oxidative stress. However, Van Duijnhoven et al. also showed that the subjects’ starting fitness levels were negatively correlated with oxidative stress, as assessed with the malondialdehyde (MDA) concentration. In contrast, Goldhardt et al. evaluated the effect of associating FES with two different exercises but carried out the FES before the training session and not during it. Goldhardt submitted participants with SCI to two different single training sessions: first, FES followed by treadmill walking with body weight support, and then FES followed by walking with a floor walker. Both acute exercises caused increased concentrations of oxidative stress markers, but only the exercise with the walker also activated an antioxidant response. This means that exercise influences oxidative stress in a protocol-dependent manner. In contrast to the findings of Van et al., Ordonez, F.J., et al. [83] (Table 3) demonstrated that (chronic) aerobic exercise, with arm-cranking, increased the total antioxidant statuses (TASs) of plasma and GPx (markers of antioxidant processes) and reduced concentrations of MDA and carbonyl groups (markers of oxidative stress) in participants with SCI. However, the number of training sessions was higher in the study by Ordonez et al. (36 sessions) than in Van Duijnhoven’s study (20 sessions), with higher intensity, and the protocol did not provide for the use of FES. However, when a single session of the same exercise at the same moderate intensity but with a longer duration (2 h instead of the 30 min for Ordonez et al.) was considered and evaluated by Mitsui et al. [84] (Table 3), the concentrations of oxidized low-density lipoprotein (oxLDL), an indicator of oxidation, did not change in subjects with SCI compared to able-bodied (AB) subjects. This seems to be associated with concentrations of adrenaline, which were higher in AB subjects in the study by Mitsui et al. Indeed, the antioxidant role of adrenaline has been already demonstrated [85]. The results suggest that increases in plasma adrenaline levels during exercise contribute to increases in plasma oxLDL levels but that subjects with SCI do not have the same response. In fact, this type of single-session exercise does not seem to alter the oxidative states of these populations. However, when the same exercise was performed to exhaustion with higher intensity in the study by Wang et al. [5] (Table 3), oxidative markers increased only in the SCI group, but the “antioxidant” defense mechanisms after this type of acute exercise were activated only in the control group (subjects without SCI). Another key issue is the association between a subject’s daily amount of physical activity and their antioxidant capacity. Inglés et al. [86] monitored the amount of moderate to vigorous physical activity (MVPA) performed by subjects with SCI for one week, dividing them successively into two groups, one active and one sedentary, and then assessing their fitness statuses with an incremental exhaustion test. They noted that both groups had significant increases in MDA and protein carbonylation after the test but only the active subjects also had increases in their concentrations of the antioxidant markers exercise-induced catalase and GPx (Table 3). However, that study assessed physical activity with only an accelerometer, not giving specific information on the type of physical or sports activity performed. Similar results have been found in tetraplegic rugby players; Hübner-Woźniak et al. [87] compared subjects with tetraplegia, who had been playing rugby for about 7 years with a biweekly frequency and a duration of two hours per training session, with inactive subjects with the same pathology (Table 3). Rugby is a sport that combines different exercise intensities, alternating high-intensity phases with low-intensity phases and also aerobic phases with anaerobic phases, also requiring skills such as speed, muscle strength, and endurance. In fact, Hübner-Woźniak et al. found that resting catalase and GPx activity concentrations were higher in tetraplegic rugby player subjects than in sedentary subjects. In contrast to the results of Hübner-Woźniak et al., Garbeloti et al. [88] analyzed the effects of wheelchair basketball, a discipline categorized among the disciplines with mixed aerobic–anaerobic effort in the same way as rugby, in subjects with SCI, comparing them to sedentary subjects with SCI and sedentary AB subjects. The wheelchair basketball players had been practicing this sport for 7 years, three times a week. However, no statistically significant differences were found in the nitric oxide (NO) concentrations of the two SCI subject groups nor in their thiobarbituric acid-reactive substance (TBARS) concentrations. Garbeloti explained these results by hypothesizing that the disuse of the lower limb muscles did not modify the production of nitric oxide or oxidative stress because it activated a protective metabolic mechanism involving vascular factors, independently from the exercises of the upper limbs. However, this point needs further clarification not specified by the authors. Therefore, wheelchair basketball seemed not to be effective in improving the antioxidative capacities of the subjects but did not alter the TBARS levels accordingly. Basketball is an activity well-tolerated by subjects with SCI because it does not produce excessive oxidative stress; the same can be said for performing a wheelchair half-marathon race. In fact, Mitsui et al. [89] analyzed the concentrations of derivatives of reactive oxygen metabolites (d-ROMs) and ox-LDL as oxidative stress markers and of biological antioxidant potential (BAP) and adrenaline as antioxidative mechanism markers before and after a wheelchair half-marathon in subjects with cervical SCI and lumbar SCI. By collecting blood samples 10  min and 1 h after the end of the competition, they found that there were no changes in the ox-LDL or d-ROMs in the two groups and that the only statistically significant differences were the increases in adrenaline and BAP, only within 10 min from the end of the race and only in the lumbar SCI group. This also underlines different responses according to the lesion level. However, in Mitsui’s study, the two groups (lumbar and cervical SCI) had different BMIs and ages, which can influence d-ROM concentration.

**Table 3:** This table shows the studies in the current literature that have analyzed the effects of physical activity on oxidative stress in subjects with SCI by reporting the characteristics of individual protocols.

Authors	Groups	Training Protocols	Training Session Duration	Training Period	Training Intensity	Fitness Level Assessment	Oxidative Stress Markers	Antioxidative Stress Markers	Techniques	Results
Van Duijnhoven et al. [80]	AB SCI	FES during leg cycle ergometer exercise	30′	20 sessions	~6.1 W at 50 rpm	Incremental maximal exercise	MDA	SOD GPx	MDA-TBA: fluorescence detection SOD: photometry GPx: spectroscopy	Correlation between fitness level and MDA. No changes in MDA, SOD, or GPx.
Goldhardt et al. [81]	SCI	FES + treadmill walking session FES + floor walker session	30′ + 60′ 30′ + 60′	Single session	4.5 km/h, as fast as they could	NS	TBARSs AOPPs Nox	CAT GSH	TBARSs: spectrofluorimetry Nitrite: spectrophotometry AOPPs: spectrophotometry CAT: plasma samples were incubated with ethanol and triton GSH: plasma incubated with DTNB, b-NADPH, and NaHCO3 as stabilizing agents and M phosphate–potassium buffer	Treadmill session: increases in AOPPs, NOx, and TBARSs without changes in antioxidant mediators. Walker training: elevations in AOPPs and NOx but also in CAT and GSH levels.
Ordonez et al. [83]	SCI	Arm-cranking	30′	36 sessions	50% to 65% of HRmax	Continuous incremental workload test to exhaustion	MDA carbonyl groups	TAS GPx	TAS: spectrophotometry MDA: fluorimetric detection Plasma carbonyl: hemolysis GPX: supernatant of erythrocyte hemolysates	TAS and GPX activity were significantly increased, with reductions in MDA and carbonyl groups.
Mitsui et al. [84]	SCI AB	Arm-cranking	2 h	Single session	60% Vo2	Progressive VO2 max test	oxLDL	Adrenaline	oxLDL: enzyme-linked immunosorbent assay Adrenaline: high-performance liquid chromatography	Increased plasma adrenaline levels in both groups but less so with SCI. Plasma oxLDL significantly increased levels only in AB subjects.
Wang et al. [90]	AB SCI	Continuous incremental workload test until exhaustion	Until exhaustion	Single session	+10 W every 2′	Continuous incremental workload test to exhaustion	urinary 8-iso-PGF2_ 11-dehydro- TXB2	NO	NO: Griess reagent-based colorimetric method 11-dehydro-TXB2: Jaffe alkaline picrate method 8-iso-PGF2_: enzyme-linked immunosorbent assay	Strenuous arm exercise increased levels of urinary 8-iso-PGF2_ and 11-dehydro-TXB2 only in the SCI group. NO increased only in AB subjects.
Inglés et al. [86]	Active SCI Non-active SCI	MVPA	>180′	7 days	>3 METS	GET until volitional exhaustion	MDA protein carbonylation	CAT GPx	MDA: chromatographic technique Protein carbonylation: immunoblot detection of plasma protein carbonyl groups Catalase and GPx mRNA: real-time PCR	Significant increases in plasma MDA and protein carbonyls after the GET, but subjects with active SCI had higher exercise-induced catalase and GPx expression than non-active subjects.
Hübner-Woźniak et al. [87]	Sedentary AB Rugby AB Rugby T Sedentary T	Wheelchair rugby	2 h 2 days/w	7.1 ± 3.4 years	NS	NS	NS	SOD CAT GPx GR8 TAS	SOD: erythrocyte hemolysates GPX: whole-blood hemolysates GR: erythrocyte hemolysates CAT: erythrocyte hemolysates CAT: standard curve TAS: ABTS	CAT and GPX activities were significantly higher in rugby players than sedentary subjects. TAS was higher in AB groups.
Garbeloti et al. [88]	Basketball SCI Sedentary SCI Sedentary AB	Wheelchair basketball	3 days/w	7 years	NS	NS	TBARSs	NO	NO: Griess nitrate reductase method TBARSs: OXltek^®^ TBARS assay	Sedentary AB group had higher levels of TBARSs. No differences were found between groups in NO concentrations.
Mitsui et al. [89]	C SCI L SCI	Wheelchair half-marathon race	NS	Single session	NS	NS	D-ROMs oxLDL	BAP Adrenaline	oxLDL: enzyme-linked immunosorbent assay Oxidative stress: d-ROM test Antioxidant abilities: BAP test Adrenaline: high-performance liquid chromatography	d-ROMs and oxLDL did not change. BAP and adrenaline increased only in LSCI.

AB: able-bodied (subjects); SCI: (individuals with) spinal cord injury; FES: functional electrical stimulation; MDA: malondialdehyde; SOD: superoxide dismutase; GPx: glutathione peroxidase; TBARSs: thiobarbituric acid-reactive substances; AOPPs: plasma advanced oxidation protein products; CAT: catalase; GSH: glutathione; NO: nitric oxide; NOx: nitrogen oxide; T: tetraplegia; GR8: glutathione reductase; TAS: plasma total antioxidant status; NS: not specified; GET: graded exercise test; d-ROMs: derivatives of reactive oxygen metabolites; oxLDL: oxidized low-density lipoprotein; BAP: biological antioxidant potential; MVPA: moderate to vigorous physical activity; HR: heart rate.

In conclusion, few studies in the literature have analyzed the effect of physical exercise on the oxidative stress of subjects with SCI (particularly, in considering the Scopus and PubMed databases, eight papers have considered the effect on oxidative stress and one has considered only the antioxidant effect). Of these, five have referred to evaluation of single sessions or single incremental tests, evaluating only the acute effect of exercise, and others have evaluated the effects of long-term training protocols (only two have evaluated sports activity protocols and only two have evaluated physical activity protocols) programmed in order to also train the antioxidant capacity in the SCI condition.

Nevertheless, from these studies, analyzed by us, we can draw the following conclusions:

- FES can be useful in programming the training plans of individuals with SCI when combined with stimuli with appropriate intensities, but the duration, intensity, and timing of administration can affect the effects on oxidative stress management;
- Twelve weeks of aerobic exercise with a three-week frequency and an intensity of about 60% of the maximum heart rate are suitable to train the antioxidant capacities of subjects with SCI;
- Exhaustion exercise seems to be poorly tolerated in subjects with SCI because they fail to have adequate antioxidant responses;
- Active (>180′ per week of MVPA) SCI subjects have greater antioxidative capacities in response to oxidative damage, induced by high-intensity to exhaustive exercise, than inactive subjects with SCI;
- The exercise intensity of wheelchair basketball is adequate and that of a half-marathon race is not excessive for trained SCI subjects because the balance between oxidation and antioxidation is maintained during these two sports activities.

## 6. Perspective

The effectiveness of exercise in the treatments of numerous diseases is now an undisputed fact. In individuals with SCI, exercise has a positive and lasting impact in both promoting neural recovery and reducing secondary complications. Indeed, exercise promotes the reconstruction of neuronal structures, cellular differentiation, and the expression of neurotrophic factors. People with SCI have shown increased risk factors (e.g., systemic inflammation, hyperinsulinemia, lipidemia, and lactate) for the development of metabolic disease [91]. Significantly, exercise improves aspects of metabolic regulation and cardiorespiratory fitness, regulates cellular electrophysiological functions, and reduces muscle imbalances by improving overall tone. It also increases the range of motion and reduces spasticity [92,93].

Exercise improves (anti)oxidative statuses in patients with SCI. It is well-known that ROSs and oxidative stress are hallmarks of secondary injury underlying SCI. However, it should be highlighted that ROSs and oxidative stress are also implicated in the growth, differentiation, and autophagy processes, which represent key mechanisms for maintaining cellular homeostasis [94]. A transitory oxidative status is needed during exercise to trigger the adaptive mechanisms of skeletal muscles [95]. Furthermore, ROS-based signaling pathways are implied in several skeletal muscle responses to exercise, such as the modulation of antioxidant enzymes, increased insulin sensitivity, mitochondrial biogenesis, and the modification/adaptation of muscle contraction [96,97,98].

Exercise is medicine and, as such, needs the right doses, to be advised and monitored by professionals. The SCI guidelines recommended for cardiorespiratory fitness and muscle strength benefits are to engage in at least 20 min of moderate- to vigorous-intensity aerobic exercise two times per week and three sets of strength exercises for each major functioning muscle group, at a moderate to vigorous intensity, two times per week. To ensure cardiometabolic health benefits, adults with SCI are prompted to perform at least 30 min of moderate- to vigorous-intensity aerobic exercise three times per week [99]. Considering that exercise can alter the balance between oxidative stress and antioxidant factors, it is necessary to create an ad hoc training program adapted to the difficulties of and capable of providing emotional support to and helping a person with SCI. 

Overall, it is possible to state that regular and adapted exercise could give benefits in managing the oxidative/antioxidative ratios of SCI patients.

## Figures and Tables

**Table 1:** American Spinal Injury Association impairment scale.

Grade	Type of Injury	Clinical Description
A	Complete	Complete injury with loss of motor and sensory functions, including sacral roots.
B	Incomplete	Incomplete injury with preserved sensory functions but complete loss of motor function below the neurological level, including the sacral segments S4–S5.
C	Incomplete	Preserved motor function below the injury level; less than half of the key muscles below the neurological level will have a muscle grade of less than 3.
D	Incomplete	Preserved motor function below the injury level; at least half of the muscles below the neurological level will have a muscle grade of 3 or more.
E	Normal	Normal motor and sensory functions.

**Table 2:** Main reactive oxygen species.

Reactive Species	Chemical Structure
Superoxide Anions	O_2_^•−^
Hydroxyl Radicals	^•^OH
Hydrogen Peroxides	H_2_O_2_
Nitric Oxide	^•^NO
Peroxynitrites	ONOO^−^
Lipid Peroxyl	LOO^−^
Lipid Alkoxyl	LO^•^
